# An Optical Fiber Sensor and Its Application in UAVs for Current Measurements

**DOI:** 10.3390/s16111800

**Published:** 2016-10-27

**Authors:** Felipe S. Delgado, João P. Carvalho, Thiago V. N. Coelho, Alexandre B. Dos Santos

**Affiliations:** Electrical Circuit Department, Federal University of Juiz de Fora, Juiz de Fora 36036-330, Brazil; felipe.souza@engenharia.ufjf.br (F.S.D.); joao.pedro@engenharia.ufjf.br (J.P.C.); alexandre.bessa@engenharia.ufjf.br (A.B.D.S.)

**Keywords:** current sensor, long-period fiber grating, optical fiber sensor, unmanned aerial vehicle

## Abstract

In this paper, we propose and experimentally investigate an optical sensor based on a novel combination of a long-period fiber grating (LPFG) with a permanent magnet to measure electrical current in unmanned aerial vehicles (UAVs). The proposed device uses a neodymium magnet attached to the grating structure, which suffers from an electromagnetic force produced when the current flows in the wire of the UAV engine. Therefore, it causes deformation on the sensor and thus, different shifts occur in the resonant bands of the transmission spectrum of the LPFG. Finally, the results show that it is possible to monitor electrical current throughout the entire operating range of the UAV engine from 0 A to 10 A in an effective and practical way with good linearity, reliability and response time, which are desirable characteristics in electrical current sensing.

## 1. Introduction

Fiber-optic sensors have acquired growing importance in the field of sensor technologies [1,2,3]. Compared with conventional devices, optical fiber sensors present many advantages [4,5]. These devices are compact, lightweight, easy to install, inexpensive and insensitive to electromagnetic interference [6], which are key features desired for sensing applications. Thus, optical fiber sensors are extremely versatile in measuring variations in temperature [7,8], strain [9], external refractive index [10], pressure [11], humidity [12] and electrical current in high voltage environments [13].

Optical current sensors using optical fibers have been investigated since the 1960s [14,15]. Since then, several researchers have proposed and demonstrated a number of practical applications using optical fiber current sensors (OFCS) [16,17,18]. One of the most popular configurations for current measurements is based on the magneto-optic effect, known as the Faraday rotation effect. For example, in [16] the authors reported an OFCS based on the Faraday effect for the measurement of plasma current in tokamaks. In [17] a long-period fiber grating (LPFG) and a Faraday effect sensing element to measure electrical current in transmission lines are described, whereas in [18], the authors have proposed the use of the Faraday rotation effect using a LPFG inscribed on a polarization-maintaining fiber as a sensor demodulator. However, these methods are affected by the fiber birefringence [5] and limited by the small Verdet constant of silica, which requires a very long fiber to enhance the sensitivity [19].

Considering these aspects, we have developed and experimentally investigated a novel OFCS based on a long-period fiber grating combined with a permanent neodymium (Nd_2_Fe_14_B) magnet attached to the LPFG sensing region. The new device presents a lightweight setup with compact size and simple installation, and provides an indirect measurement of the electrical current of an UAV engine. When the controller activates the drone engines, the battery will supply each one of the four motors according to the desired flight speed and direction. Thus, electrical current flowing within the conductor wire generates a magnetic force that deflects the permanent magnet and deforms the LPFG, which are effective external deformation sensors [9,20,21]. Therefore, according to the different shifts in the resonant bands of the LPFG transmitted spectrum, it is possible to detect the current demanded by the aircraft engine.

The experimental investigation was carried out under room temperature (27 °C) with the UAV fixed on the lab bench. Further, the electrical current variations were measured for a single UAV engine, while all the motors were spinning at the same time. Contrary to the laboratory environment, a complete version of the OFCS would be embedded into the drone, consisting of a simple scheme of a LED light source, the proposed OFCS, an optical filter and photodetectors, since the experimental tests were performed with a broadband light source and an optical spectrum analyzer in the lab.

## 2. Unmanned Aerial Vehicle Characteristics

In order to operate remotely the UAV, the user has to send command signals to the aircraft, and thus regulate the rotation speed of the four engines of the UAV. In other words, the operator regulates the pulse-width modulation (PWM) through a wireless radio-control at 2.4 GHz radio frequency (RF), indicating the desired command. The PWM signals do not act directly on the UAV engines. Actually, the flight control unit (FCU), within the aircraft, receives the PWM signals and transmits them to an electronic speed control module (ESC). The ESC module is powered by the battery of the UAV, and controls the rotation of the engine for the desired flight conditions.

**Figure 1:**
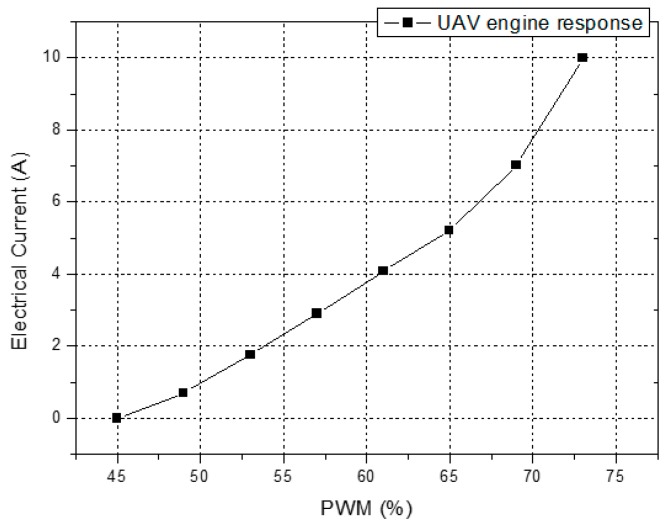
Measured electrical current demanded by the UAV engine under test as a function of different duty cycles of the PWM signals.

Figure 1 shows the detailed relationship between the duty cycle of the PWM signals and the electrical current demanded by a single engine of the UAV, measured by a conventional clamp ammeter. The UAV engine demands more electrical current in high duty cycles, thus its response is not linear throughout the entire operation range and it reaches its rotation speed limit when the PWM signal has a duty cycle of 73%, which corresponds to 10 A.

In addition to the 2.4 GHz RF transmission of the pilot signals to the aerial vehicle, more communications technologies are embedded into the UAV system, such as the global positioning system (GPS), onboard WiFi signals transferring video signals back to the pilot, and telemetry. There is also noise that may cause electrical magnetic interference (EMI) generated by the motors of the aircraft and ESCs as well. Thus, considering these aspects, the use of an OFCS provides electrical current measurements with reliability, due to its immunity to EMI from the UAV components and insensitivity to entangled frequencies in the same band, which conventional sensors do not provide.

## 3. Theory

Long-period gratings (LPFG) are fiber-optic devices that consist of a periodic modulation of the optical fiber properties, which is normally a perturbation of the refractive core index of a fiber section [22]. Several techniques have been reported concerning the fabrication process of the gratings, such as exposure to ultraviolet (UV) irradiation [22], irradiation by femtosecond pulses in the infrared (IR) [23], irradiation by CO_2_ lasers [24], as well as ion beam implantation, etching, mechanical arrangements, and electric arc discharges [25,26,27,28]. Besides, the LPFG usually has a period (Λ) of 100 µm to 1000 µm and the grating promotes coupling between the propagating core mode (LP_01_) and the *m* different co-propagating cladding modes (LP_0m_). The result of the coupling between propagating and co-propagating modes that can be observed in the transmission spectrum of the LPFG is a high attenuation of the cladding modes, represented by a series of attenuation resonance bands according to the following equation [6,29]:

(1) $$\beta_{co}-\beta_{cl}^{m}=\frac{2\pi }{\Lambda }$$

where $\beta_{co}$ and $\beta_{cl}^{m}$ are the propagation constants of the core and the *m*th-order cladding modes, respectively, and Λ is the grating period. The propagation constant can be rewritten as 2π*n_eff_*/λ, being *n_eff_* and λ the effective refractive index and the light wavelength, respectively. Thus, from Equation (1) the resonance phase matching condition can be easily determined as [30]:

(2) $$\lambda_{m}=\left(n_{co}-n_{cl}^{m}\right)\Lambda$$

where *λ_m_* is the resonance wavelength of the *m*th-order cladding mode, and *n_co_* and $n_{cl}^{m}$ are the effective refractive indices of the fundamental core mode and the *m*th-order cladding mode, respectively. Changes in the temperature, strain, refractive index of the external medium surrounding the LPFG sensor can alter the grating period as well as the differential refractive index of the core and cladding modes and thus, lead to variations in the resonance condition due to the rejection wavelengths (*λ_m_*) dependence to external parameters variations [31,32,33]. Therefore, it is possible to extract the environmental information from the analysis of the shifts occurring at the *λ_m_* parameter.

## 4. Experimental Setup and Sensing Principle

**Figure 2:**
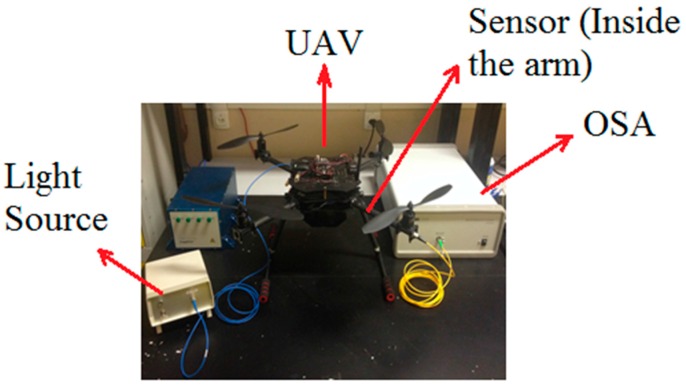
Experimental setup for UAV current sensing.

The experimental setup is shown in Figure 2. It consists of a broadband light source (BBS), an optical spectrum analyzer (OSA, Thorlabs Fourier Transform Spectrometer, Thorlabs, Newton, NJ, USA), the proposed OFCS (LPFG + permanent magnet) placed inside the arm of the vehicle, and the UAV. 

**Figure 3:**
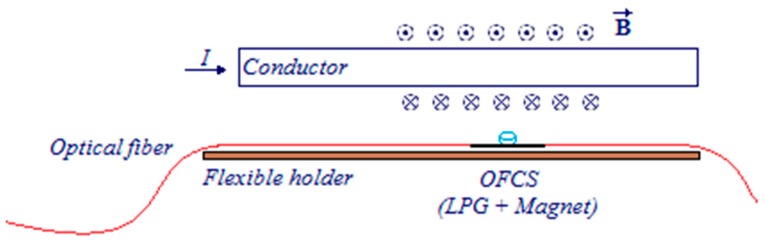
Schematic representation of the proposed optical fiber current sensor (OFCS) combining a LPFG with a neodymium permanent magnet.

The sensing device is obtained fixing with an epoxy resin a neodymium (Nd_2_Fe_14_B) magnet, the LPFG and the flexible holder structure. This configuration, shown in Figure 3, provides unidirectional displacements to deform the LPFG sensing head. Further, the proposed sensor is compact, lightweight and easy to install, these characteristics allow it to be easily employed according to any UAV features, such as conductor wires, ESCs and engines position and size. Possible displacements in other directions can be mitigated using another grating without the magnet as can be seen in literature [34].

**Figure 4:**
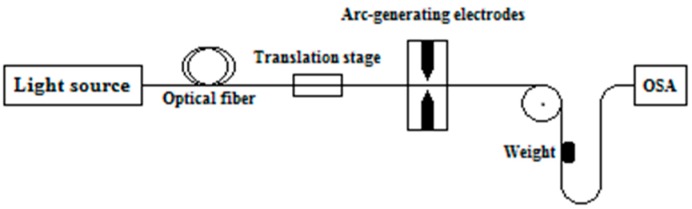
Fabrication scheme used for fabrication of the LPFGs.

**Figure 5:**
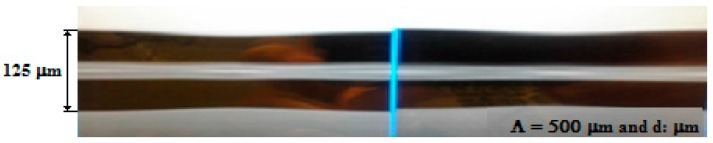
Microphotograph of the side view of the long-period grating sensor produced by arc discharges.

An LPFG sensor was fabricated in the Laboratory of Instrumentation and Telemetry (LITel) at Federal University of Juiz de Fora (UFJF). Figure 4 shows the fabrication scheme of the LPFG sensors. It was manufactured in a single-mode optical fiber (SMF28) using the electric arc discharges method [25,35]. The fiber was arc-induced using a fusion splicer (KL-300T, Jilong, Nanjing, China) with electrical arc discharges of 20 bits and 470 ms, grating period of Λ = 500 µm and constant external tension of 2 g weight [28]. During the fabrication process, an optical spectrum analyzer was used to monitor the transmission spectrum characteristics. Further, these fabrication parameters were chosen in order to achieve coupling of the highest possible cladding mode near λ = 1550 nm, which would provide higher sensitivity to external parameters sensing [30,31]. The LPFG sensor microphotograph after the manufacturing process is shown in Figure 5. There is a periodical diameter reduction and elongation of the fiber, which, actually, were introduced by the tapering geometric deformation after the arc discharges [36].

The neodymium magnet present in the LPFG sensing region can be attracted or repelled depending on the position of its magnetic poles. In the experiment, we have placed the magnet with the LPFG in order to provide a repulsive electromagnetic force, due to the electrical current variations in the conductor wire of the UAV engine. The force repels the magnet and thus, causes lateral deformation on the LPFG sensor, leading to displacements of the resonant attenuation band of the LPFG.

Finally, it is possible to measure indirectly the electrical current demanded by the UAV engine according to the desired PWM control signals. This is the basic principle of the proposed optical fiber current sensor. 

## 5. Experimental Results

**Figure 6:**
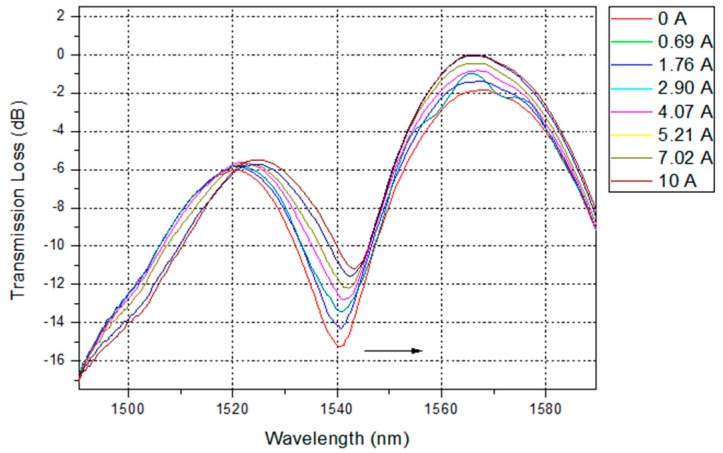
Transmission loss spectra of the LPFG sensor under different electrical current demanded by the UAV engine.

The response of the current sensor was experimentally investigated by applying PWM duty cycles in the range from 45% to 73%, which corresponds to an electrical current of 0–10 A supplied to a single UAV motor, as shown in Figure 1. We measured and recorded the transmission spectrum of the LPFG sensor during the process of the experiment, and it presented a significant change in the transmission, due to the electrical current variation, as shown in Figure 6.

**Figure 7:**
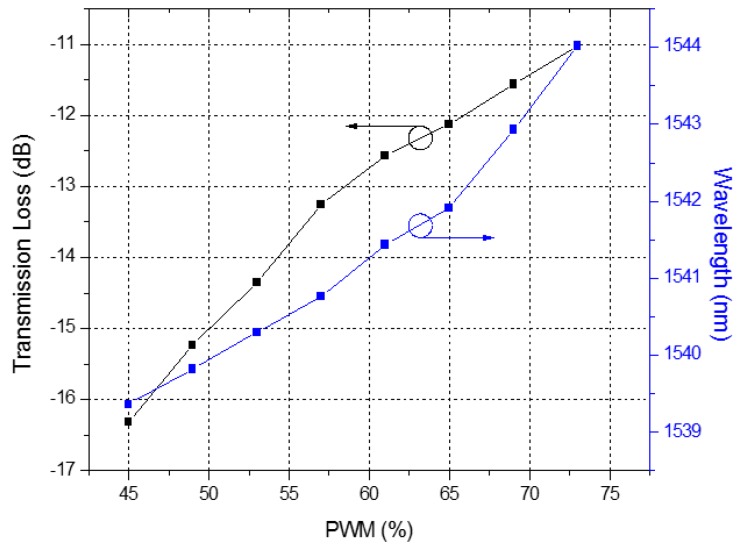
Dip wavelength and transmission loss versus PWM control signals.

**Figure 8:**
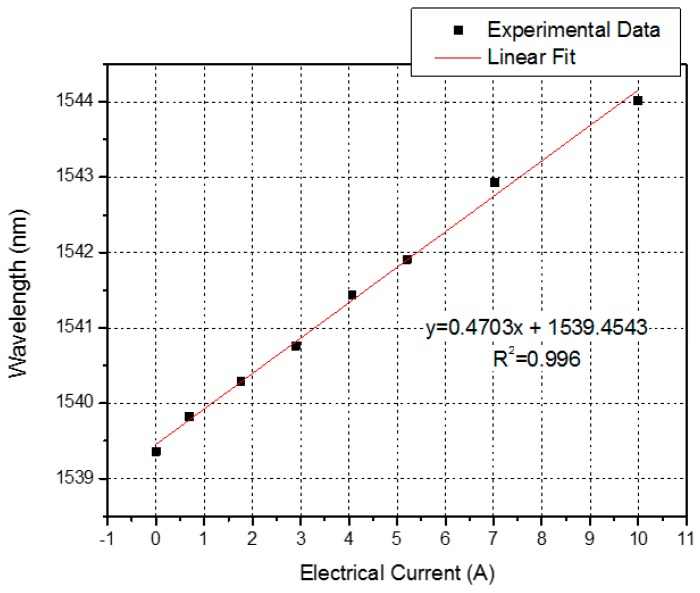
Experimental data fit of the dip wavelength as a function of electrical current.

**Figure 9:**
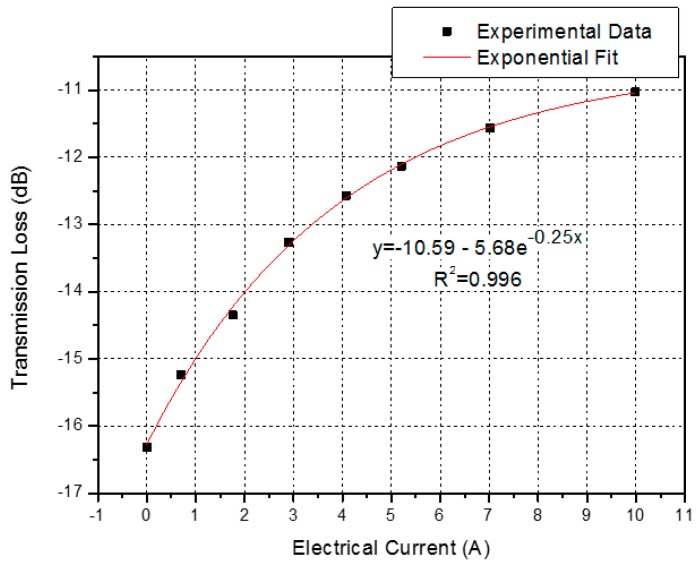
Experimental data fit of the transmission loss as a function of the electrical current.

The attenuation band shifted from 1539.36 nm to 1544.02 nm, with a spectral variation shift of 4.66 nm. In addition, the dip of the transmission loss shifted from −16.32 dB to −11.03 dB, varying 5.29 dB. We can see the detailed response curves of the LPFG for the PWM signals in Figure 7, and its corresponding wavelength and intensity responses for electrical current in Figure 8 and Figure 9, respectively. In Figure 7, we observe that the response for PWM duty cycle signals presents the same behavior as the electrical current variation, because of their correlation, as shown in Figure 1. As the electrical current demanded by the UAV engine increased, the dip wavelength shifted towards higher wavelengths. By data fitting, the sensitivity of 0.4703 nm/A was achieved with high degree of electrical current linearity, confirmed by the R^2^ coefficient derived from the fitting process, as seen in Figure 8.

On the other hand, Figure 9 shows the transmission loss decreasing as the electrical current increases. Furthermore, this intensity changes behavior can be fitted well by an exponential function with a R^2^ coefficient value of 0.996 over the sensing range. The exponential curve in Figure 9 can be used to calibrate the nonlinearity between the transmission loss and the electrical current variation for the OFCS. In addition, the behavior of the transmission loss agrees well [23], and is based on the periodic refractive index modulation caused by the external deformation. When the LPFG is placed under deformation, it generates a periodic strain field owing to the expanded cross section area, and thus affects the refractive index periodic distribution of the optical fiber and produces an attenuated loss dip in the spectrum, as shown in Figure 6.

## 6. Conclusions

We have proposed and experimentally investigated a simple and effective optical fiber sensor for UAV current monitoring. The novel sensor based on long-period fiber gratings combined with a permanent neodymium magnet provides a practical way to measure indirectly the electrical current flowing within the conductor wire of the UAV engine. The electrical current sensing has been achieved successfully by measuring the displacements of LPFG spectrum based on wavelength shifts or intensity changes with the entire UAV operation range from 0 A to 10 A, as observed in Figure 8 and Figure 9.

The results showed a good wavelength linear sensitivity of 0.4703 nm/A, observed in Figure 8. Besides, using the proposed method, we can also monitor the electrical current in the UAV engine based on intensity changes in the transmitted spectrum, as shown in Figure 9.

Finally, we can extend the proposed method to a portable OFCS system and thus, monitor electrical current in every engine of the UAV, since there is no concerns about EMI from the aircraft devices using the proposed OFCS measurement scheme. The portable sensor system would be embedded into the UAV, and would consist of a simple scheme of a LED light source, the proposed OFCS, an optical filter, photodetectors and a simple electronic unit.
